# Netrin-1 is a novel regulator of vascular endothelial function in diabetes

**DOI:** 10.1371/journal.pone.0186734

**Published:** 2017-10-23

**Authors:** Haroldo A. Toque, Aracely Fernandez-Flores, Riyaz Mohamed, Ruth B. Caldwell, Ganesan Ramesh, R. William Caldwell

**Affiliations:** 1 Department of Pharmacology & Toxicology, Medical College of Georgia, Augusta University, Augusta, GA, United States of America; 2 Vascular Biology Center, Medical College of Georgia, Augusta University, Augusta, GA, United States of America; 3 Charlie Norwood VA Medical Center, Augusta, Georgia, United States of America; University of Southampton, UNITED KINGDOM

## Abstract

**Background:**

Netrin-1, a secreted laminin-like protein identified as an axon guidance molecule, has been shown to be of critical importance in the cardiovascular system. Recent studies have revealed pro-angiogenic, anti-apoptotic and anti-inflammatory properties of netrin-1 as well as cardioprotective actions against myocardial injury in diabetic mice.

**Aim:**

To examine the role of netrin-1 in diabetes-and high glucose (HG)-induced vascular endothelial dysfunction (VED) using netrin-1 transgenic mice (Tg3) and cultured bovine aortic endothelial cells (BAEC).

**Main outcome:**

Overexpression of netrin-1 prevented diabetes-induced VED in aorta from diabetic mice and netrin-1 treatment attenuated HG-induced impairment of nitric oxide synthase (NOS) function in BAECs.

**Methods and results:**

Experiments were performed in Tg3 and littermate control (WT) mice rendered diabetic with streptozotocin (STZ) and in BAECs treated with HG (25 mmol/L). Levels of netrin-1 and its receptor DCC, markers of inflammation and apoptosis and vascular function were assessed in aortas from diabetic and non-diabetic Tg3 and WT mice. Vascular netrin-1 in WT mice was reduced under diabetic conditions. Aortas from non-diabetic Tg3 and WT mice showed similar maximum endothelium-dependent relaxation (MEDR) (83% and 87%, respectively). MEDR was markedly impaired in aorta from diabetic WT mice (51%). This effect was significantly blunted in Tg3 diabetic aortas (70%). Improved vascular relaxation in Tg3 diabetic mice was associated with increased levels of phospho-ERK1/2 and reduced levels of oxidant stress, NFκB, COX-2, p16^INK4A^, cleaved caspase-3 and p16 and p53 mRNA. Netrin-1 treatment prevented the HG-induced decrease in NO production and elevation of oxidative stress and apoptosis in BAECs.

**Conclusions:**

Diabetes decreases aortic levels of netrin-1. However, overexpression of netrin-1 attenuates diabetes-induced VED and limits the reduction of NO levels, while increasing expression of p-ERK1/2, and suppressing oxidative stress and inflammatory and apoptotic processes. Enhancement of netrin-1 function may be a useful therapeutic means for preventing vascular dysfunction in diabetes.

## Introduction

Diabetes is growing in prevalence and responsible for many cardiovascular problems among its millions of sufferers worldwide [1,2]. The vascular endothelium is a key regulator of vascular smooth muscle tone through production of the vasodilator nitric oxide (NO). Vascular endothelium dysfunction (VED) is a critical and initiating factor in the development of the vascular complications induced by diabetes [1,3–5]. NO is synthesized from L-arginine by NO synthase (NOS). Diabetes-induced reduction of L-arginine availability via increased arginase activity can cause NOS uncoupling, excessive generation of reactive oxygen species (ROS), reduced NO levels and VED [6,7]. Better understanding of signaling mechanisms that increase vascular NO levels is needed to prevent or reverse VED.

Earlier studies have shown that netrin-1, a secreted axon guidance molecule [8,9], strongly stimulates NO production to promote vascular and cardiac endothelial cell migration and proliferation [10,11]. While at least eight netrin receptors have been characterized in the vascular system, netrin-1 function is mainly mediated by two families of receptors: DCC (deleted in colorectal cancer) and UNC5 (uncoordinated-5) families [12,13]. Binding of netrin-1 to DCC exerts important suppression of apoptosis [14,15], tumorigenesis [16] and angiogenesis [10,17]. Netrin-1 induced NO production is DCC-dependent, and NO causes a feed-forward increase in DCC expression and subsequent activation of the mitogen-activated protein kinase ERK1/2 [10,11]. Exogenous administration of netrin-1 has shown cardioprotective effects against ischemia/reperfusion (I/R) injury, via an increase in NO that is dependent on the DCC/ERK1/2/eNOS/DCC feed-forward pathway [11]. Netrin-1 also ameliorates myocardial infarction in a diabetic animal model [18] and abrogates I/R-induced cardiac mitochondrial dysfunction via NO-dependent attenuation of NADPH oxidase isoform 4 activity and recoupling of NOS [19]. Additionally, in I/R injury of the kidney, downregulation of netrin-1 enhances inflammatory processes whereas overexpression of netrin-1 induces vascular regeneration and suppresses inflammation and apoptosis in diabetic nephropathy [20]. Further, netrin-1 treatment has been shown to abolish autophagy which occurs in a coronary ligation model of myocardial infarction [21].

Considering the cardioprotective and beneficial impact of netrin-1 in myocardial infarction, we hypothesized that overexpression of netrin-1 in mice will prevent diabetes-induced VED by increasing NO production, limiting oxidative stress and preventing activation of inflammatory and apoptotic mediators through the DCC-ERK1/2 pathway. We also examined the endothelial-specific effects of netrin-1 on NO production and apoptosis pathways by experiments using bovine aortic endothelial cells (BAECs) exposed to high glucose (HG, 25 mmol/L).

## Material and methods

### 2.1 Animals and tissue preparation

Protocols were conducted in accordance with the rules of the Augusta University’s Animal Use for Research and Education Committee (approval no BR10-02-297). Netrin-1 transgenic (Tg3) mice were created as described before [22]. Briefly, chicken netrin-1 was cloned upstream of the L-fatty acid binding protein (FABP) promoter and used for microinjection. FABP promoter leads the enhancement of the transgene in the gut, but subsequent screening revealed promoter activity and transgene expression in other tissues including the small intestine, colon, spleen, brain and kidney [20].

Experiments were performed in Tg3 and their wild type (WT) littermate mice eight weeks after diabetes was induced by streptozotocin (STZ). Diabetic (D) mice received intraperitoneal (ip) injections of STZ (50 mg/kg) every other day for up to 3 injections. Control non-diabetic (ND) mice were given vehicle (citrate buffer). Mice with blood glucose levels >350 mg/dL were considered diabetic. Body weight and glucose levels of each mouse were measured at the time of injections and eight weeks after treatment. Two days before ending the 8-weeks period after STZ treatment, serum insulin levels were obtained by tail-vein bleeds from overnight-fasted or 2 hr after feeding in overnight-fasted mice. Mice were anesthetized with a mixture of ketamine/xylazine (100/10 mg kg^-1^, i.p.), blood samples were collected and aortas were rapidly excised and placed in chilled Krebs solution.

### 2.2 Vascular functional studies

Aortic rings were mounted in myograph organ bath chambers (Danish Myograph Technology, Aarhus, Denmark). Vessels were adjusted to maintain a passive tension of 5 milliNewtons (mN) and equilibrated for 60 min in physiological saline solution of the following composition (in mmol/L: NaCl, 118; NaHCO_3_, 25; glucose, 5.6; KCl, 4.7; KH_2_PO_4_, 1.2; MgSO_4_ 7H_2_O, 1.17 and CaCl_2_ 2H_2_O 2.5) at 37°C and continuously bubbled with 5% CO_2_ and 95% O_2_. Changes in isometric force were recorded in a PowerLab 8/SP data acquisition system (software Chart 5.0; AD Instruments, Colorado Springs, CO, USA). After the equilibration period, vessels were exposed to a potassium solution (KCl, 80 mmol/L) to assess arterial contractile function.

After washing out KCl, cumulative concentration-response curves to acetylcholine (ACh, an endothelium-dependent vasodilator; 0.001–10 μmol/L) were obtained in aortic rings from non-diabetic and diabetic WT and Tg3 mice after pre-contraction with phenylephrine (PE, α_1_-adrenergic receptor agonist; 0.1 μmol/L for diabetic and 1 μmol/L for non-diabetic vessels). Also, cumulative concentration-response curve to sodium nitroprusside (SNP, a NO donor; 0.0001–10 μmol/L) or PE (0.001–10 μmol/L) were performed in vessels from non-diabetic and diabetic WT and Tg3 mice.

### 2.3 Quantification of mRNA by real-time RT-PCR

RNA was isolated using TRIzol reagent (Life Technologies, Grand Island, NY). Real-time RT-PCR was performed in an ABI 7700 sequence detection system (Applied Biosystems; Life Technologies, Foster City, CA). Total RNA (3 μg) was reverse transcribed in a reaction volume of 40 μL using an Omniscript RT kit (Qiagen, Valencia, CA) and random primers. The product was diluted to a volume of 150 μL, and 5 μL aliquots were used as templates for amplification using SYBR Green PCR amplification reagent and gene-specific primers. The following primer sets were used: netrin-1 (forward: 5-AAGCCTATCACCCACCGGAAG-3; reverse: 5-GCGCCACAGGAATCTTGATGC-3); p53 (forward: 5-GAGAGACCGCCGTACAGAAG-3; reverse: 5-AGCAGTTTGGGCTTTCCTCC-3); p16 (forward: 5-CGAACTCGAGGAGAGCCATC-3; reverse: 5-TACGTGAACGTTGCCCATCA-3); bos taurus netrin-1 (forward: 5-CTGGCAGTCGGAGAACTACC-3; reverse:5-CAGAACTGCAGGCTCACGTA-3); DCC (forward: 5-TCTTCACAGGATTGGAGAAAGGC-3; reverse: 5-GAGGAGGTGTCCAACTCATGATG-3). The amount of DNA was normalized to the ß-actin (forward: 5-AGAGGGAAATCGTGCGTGAC-3; reverse: 5-CAATAGTGATGACCTGGCCGT-3); mouse HPRT (forward: 5-GAAAGACTTGCTCGAGATGTCATG-3; reverse: 5-CACACAGAGGGCCACAATGT-3); or bos taurus 18S (forward: 5-AGAGGGAAATCGTGCGTGAC-3; reverse: 5-CAATAGTGATGACCTGGCCGT-3) signal amplified in a separate reaction.

### 2.4 Cell culture and treatment

Bovine aortic endothelial cells (BAECs) were used between passages 4–6. Upon 80% confluence, cells were placed in M-199 growth medium (Invitrogen) containing normal glucose (NG, 5.5 mmol/L) or high glucose (HG, 25 mmol/L), 0.2% fetal bovine serum, 50 μmol/L _L_-arginine, 100 U/ml penicillin, 100 μg/ml streptomycin and _L_-glutamine for periods of 15, 30, 45, and 60 min or 24 and 48 hrs. Another group of cultures was pretreated with netrin-1 (100 ng/ml) for 1 hr before the addition of NG, HG or mannitol as osmotic control medium (5.5 mmol/L D-Glucose + 19.5 mmol/L mannitol) for periods of 1 to 48 hrs. At the end of the treatment periods, cells were harvested for enzymatic assay and western blot analysis.

### 2.5 Western blot analysis

Protein was extracted from aorta or BAECs by lysis using 1x RIPA buffer (Upstate, Temecula) containing protease inhibitors and phosphatase inhibitors. Lysate was centrifuged at 14,000 g for 10 min at 4°C and supernatant was used for protein estimation. Equal amount of protein (20 μg) was separated by electrophoresis on a 10% SDS-polyacrylamide pre-cast gel and transferred to polyvinylidene difluoride membrane. Blots were blocked using 2% bovine serum albumin (Sigma), incubated with their respective primary antibodies (anti-arginase 1, 1:10000; anti-arginase 2, 1:250; DCC, 1:500; anti-netrin-1, 1:500, Santa Cruz Biotechnology, Inc; anti-COX2, 1:1000, Abcam; anti- β-actin, 1:5000, Sigma-Aldrich; anti-p44/42 MAPK ERK1/2 and anti-phospho p44/42/MAPK ERK1/2 at Thr^202^ and Tyr^204^, 1:1000; anti-eNOS and anti-phospho-eNOS at Ser^1177^, 1:1000; cleaved caspase-3, 1:1000; anti-NFkβ p65, 1:1000, Cell Signaling Technology, Inc) overnight at 4°C. After incubation with secondary antibodies, signals were visualized using an enhanced chemiluminescence kit (Amersham, Piscataway, NJ, USA). Bands were observed using Kodak image analyzer or Gene Snap (Syngene, Frederick, MD). Densitometric analysis was carried out using the Gene Snap software, results normalized to β-actin protein and expressed as arbitrary unit.

### 2.6 Arginase activity levels

Arginase activity was assayed by measuring urea produced from _L_-arginine as previously described [23]. Cells were lysed or frozen mouse aortas were homogenized by pulverization with 1:4 wt:vol of Tris buffer (50 mmol/L Tris-HCl, 0.1 mmol/L EDTA and EGTA, pH 7.5) containing PMSF, protease inhibitors, phosphatase inhibitors and homogenized on ice. The homogenate was sonicated and centrifuged at 12,000 g for 10 min at 4°C and supernatant was used for enzyme assay. 25 μL of cell lysate or tissue supernatant was heated with 25 μL of MnCl_2_ (10 mmol/L) for 10 min at 56°C to activate arginase. The mixture was then incubated with 50 μL _L_-arginine (0.5 M, pH 9.7) for 1 hr at 37°C to hydrolyze the _L_-arginine. The hydrolysis reaction was stopped with acid, and the mixture was then heated at 100°C with 25 μL α-isonitrosopropiophenone (9% α-ISPF in EtOH) for 45 min. Samples were kept in dark for 10 min and absorbance was then measured at 540 nm. Enzyme activity units were normalized to protein to obtain specific activity.

### 2.7 NO Levels and production in endothelial cells and aorta

Media was collected from cultures treated with NG, HG, or mannitol at 24 and 48 hr and then measured by NO-specific chemiluminescence in a Sievers 280i NO Analyzer. Other groups of samples was pre-treated with netrin-1 (100 ng/ml) for 1h before the exposure to NG or HG. Briefly, 20 μl of medium was injected in glacial acetic acid containing sodium iodide in the reaction chamber. NO_2_ is quantitatively reduced to NO under these conditions, which was quantified by NO-specific chemiluminescence detector after reaction with ozone.

Intracellular NO levels in aorta from non-diabetic or diabetic Tg3 and WT mouse and from cells exposed to NG or HG medium in the absence or presence of netrin-1 (100 ng/ml for 48 h) were measured using the NO-specific fluorescent dye 4,5-diaminofluorescein diacetate (DAF-2 DA, Calbiochem, USA). Thereafter, aortic slides or cells were stained with oxygenated DAF-2 DA solution (10 μmol/L) in the darkness and shaking water bath at 37°C for 30 min. Some of those samples were pre-treated with L-NAME (0.1 mmol/L) for 30 min before adding DAF-2 DA and used as negative controls. Fluorescence was monitored using fluorescence microscope (excitation, 488 nm; emission, 610 nm) at 10 or 20 × magnification. The fluorescent intensity was quantified using Image J software. Results were presented as mean fluorescent intensity per field and expressed as percent of control.

### 2.8 Reactive oxygen species (ROS) formation in endothelial cells and aorta

The oxidative fluorescent dye dihydroethidine (DHE) was used to evaluate *in situ* formation of ROS. Treated cells culture or aortic sections of 10-μm-thick were exposed with DHE (5 μmol/L) and incubated in a light-protected humidified chamber at 37°C for 30 min. Cell nuclei were stained with 4',6-diamidino-2-phenylindole (DAPI, blue staining). Some cells or aortic samples were incubated with the eNOS inhibitor (L-NAME, 100 μmol/L) or the NADPH oxidase inhibitor (apocynin, 10 μmol/L) for 30 min before incubation with DHE. The level of ROS was determined using fluorescence microscope (excitation, 640 nm; emission, 665 nm) at 10 or 20 × magnification. Nine pictures were captured in each well (n = 4), and the fluorescent intensity was quantified using NIH Image J software. Results were presented as mean fluorescent intensity in each field and expressed as percent of NG-treated cells.

### 2.9 Caspase-3 activity and flow cytometry analysis of endothelial apoptosis

Cells were lysed in caspase assay buffer and aliquots of 50 μg of lysate were incubated with 50 μl of 2x reaction buffer containing 10 mmol/L DTT. Then, caspase-3 substrate DEVD-*p*NA (BioVision, Milpitas, CA) was added and incubated at 37°C for 90 min. The caspase-3 activity from cells exposed to NG and HG (48 hr) in the absence or presence of netrin-1 (100 ng/ml) was quantified by the *p*NA light emission using a spectrophotometer at 405 nm.

Flow cytometry analysis was performed in BAECs exposed to NG and HG for 48 hrs. Cells were treated with netrin-1 (100 ng/ml) before incubation with HG followed by annexin-V FITC and propidium iodide (PI) (Invitrogen) staining. Apoptotic (annexin-V^+^) cells were analyzed with flow cytometry. At least 10,000 events were collected. Data were analyzed with CellQuest v3.3 software (BD Bioscience) as instructed.

### 2.10 Measurement of malondialdehyde (MDA) content in aorta and plasma

Malondialdehyde (MDA) is a stable metabolite of the ROS-mediated lipid peroxidation cascade, often used as index of oxidative stress. Measurements of lipid peroxidation were made using the TBARS assay, which is used to quantify the colorimetric reaction of the lipid peroxidation product malondialdehyde (MDA) with thiobarbituric acid (TBA). The reaction produces a colored compound which absorption is determined spectrophotometrically at λ = 532 nm. Results are expressed as μg/mg of protein.

### 2.11 Statistical analysis

Experimental values of relaxation or contraction were calculated relative to the maximal changes from the contraction produced by PE and KCl, respectively, taken as 100% in each tissue. Data were expressed as the mean ± S.E.M. Curves were fitted to all the data using nonlinear regression, and half-maximum response (pEC_50_) of each drug expressed as–log molar (M) was used to compare potency. Student’s t-test or analysis of variance (ANOVA) followed by Bonferroni post hoc test was used to evaluate the differences among the groups as appropriate. *P* < 0.05 was considered significant. Statistical analysis was undertaken using GraphPAD Prism Software Inc.

## Results

### 3.1 Animal studies

#### 3.1.1 Body weight and plasma glucose levels

**Fig 1:**
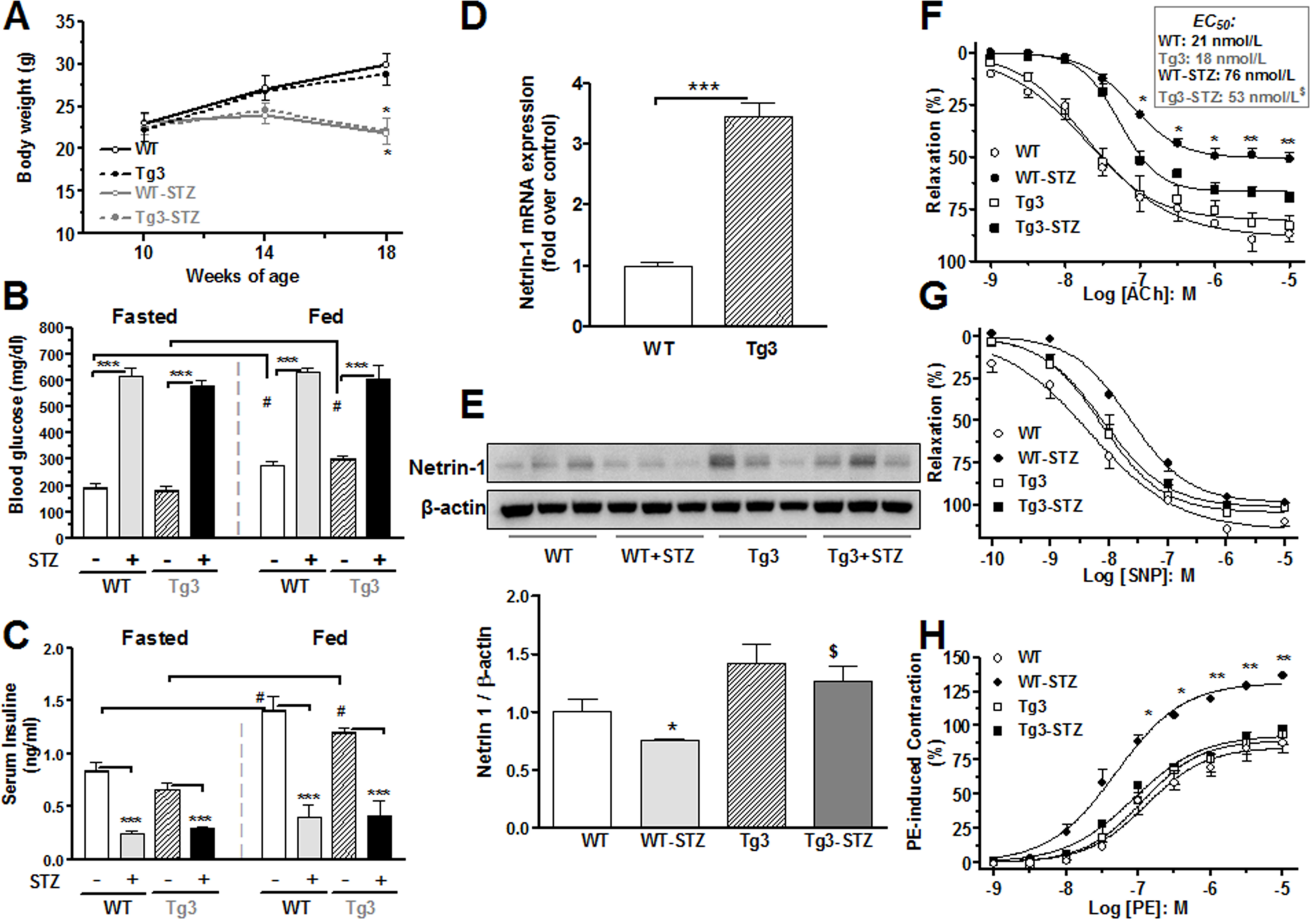
Metabolic parameters, netrin-1 expression and vascular functional studies. (**A**) Body weights of netrin-1 transgenic (Tg3) and wild type (WT) non-diabetic and diabetic mice were measured throughout the study. (**B**) Fasting and fed blood glucose and (**C**) serum insulin levels were measured in Tg3 and WT mice two days before 8-weeks of STZ treatment. (**D**) Netrin-1 mRNA and (**E**) protein expression was measured in non-diabetic or diabetic aortas from WT and Tg3 mice. EC_50_ values and concentration-response curve to acetylcholine (ACh, 0.001–10 μmol/L; **F**), sodium nitroprusside (SNP, 0.0001–10 μmol/L; **G**), and phenylephrine (PE, 0.001–10 μmol/L; **H**) were performed in non-diabetic and diabetic aortas from Tg3 and WT mice. Data are mean ± S.E.M. of 6–7 experiments. *P < 0.05; **P < 0.01; ***P < 0.001, compared with non-diabetic WT group; ^#^*P* < 0.05, compared with fasted non-diabetic WT or Tg3 group; ^$^ compared with diabetic WT mice.

Diabetes is characterized by increased blood glucose and reduced body weight in humans and animal models. We assessed the body weight and glucose levels before and every four weeks during the STZ treatment in netrin-1 transgenic (Tg3) and littermate control (WT) mice. We observed gain of body weight in WT and Tg3 non-diabetic mice, whereas diabetes induced decreases in body weights that were similar in both groups (~29%) compared with their respective control groups (Fig 1A). Diabetes induced increases in fasted blood glucose levels in both WT and Tg3 mice (~250% *versus* their control mice; Fig 1B).

#### 3.1.2 Metabolic studies

To assess the metabolic phenotype of mice, glucose levels and insulin tolerance tests are methods commonly used. These measurements were performed in blood or serum collected from WT and Tg3 mice immediately after an overnight fast or 2 hr after feeding following an overnight fast. Diabetic WT or Tg3 mice fed with chow-diet for 2 hr after overnight fasting showed elevation of glucose levels similar to those of fasted diabetic WT and Tg3 mice (Fig 1B). Also, 2-hr fed non-diabetic WT and Tg3 mice had significantly increased glucose levels *versus* their fasted control mice (~50% and ~65%, respectively).

Additionally, diabetes induced similar reductions of serum insulin levels in WT and Tg3 mice as compared with their respective controls (~82% and ~60%, respectively, Fig 1C). The 2-hr chow-diet feeding of overnight-fasted WT and Tg3 mice resulted in similar increases in serum insulin levels in non-diabetic WT (~68%) or Tg3 (~79%) *versus* with their respective fasted control. However, in diabetic WT or Tg3 mice, serum insulin levels were not altered after the 2 hr feeding as compared with the overnight fasted mice (Fig 1C).

#### 3.1.3 Netrin-1 is expressed in the aorta and its levels are decreased in diabetes

To assess netrin-1 expression in aorta from WT and Tg3 mice, we used RT-PCR and western blot analysis. We observed that aortic netrin-1 mRNA levels were much higher in control Tg3 mice (~244%) than in control WT mice (Fig 1D). Aortas from diabetic WT mice showed lower protein levels of netrin-1 (~24%) compared with non-diabetic WT mice (Fig 1E). Netrin-1 levels in diabetic Tg3 mice were lower than in non-diabetic Tg3 mice. A representative blot of three samples per group is shown in Fig 1E, but the quantification analysis was performed in 6 or 7 samples per group.

#### 3.1.4 Netrin-1 overexpression limits impairment of endothelium-dependent vasorelaxation and prevents augmented contractile response in diabetes

Endothelial dysfunction occurs in diabetes. We assessed aortic endothelial function using vascular myography. Cumulative addition of acetylcholine (ACh) to aortic rings pre-contracted with phenylephrine (PE) produced concentration-dependent relaxation in non-diabetic (WT, Tg3) and diabetic (WT-STZ, Tg3-STZ) mice (Fig 1F). Similar response of maximal relaxation (E_max_) and sensitivity (EC_50_) values were observed in vessels from the non-diabetic WT and Tg3 mice. However, maximum vasorelaxation was markedly impaired in vessels from diabetic WT (E_max_: 51±3%) compared with that of non-diabetic WT mice (E_max_: 87±4%). Diabetes markedly reduced the EC_50_ for ACh in aortas of WT mice from 21 to 76 nmol/L, but this reduction was significantly attenuated between the Tg3 groups (from 18 to 53 nmol/L in non-diabetic and diabetic group, respectively). The EC_50_ values for diabetic aorta from WT and Tg3 mice, were significantly different (Fig 1F). Further, the vasorelaxion of Tg3 diabetic aorta was partially protected against the effects of diabetes (E_max_: 70±3%) (Fig 1F).

Maximal relaxation responses to the NO donor sodium nitroprusside (SNP), an endothelium-independent relaxation agent, were not different among the groups (Fig 1G). However, EC_50_ values for SNP were increased in aortas from diabetic WT (20 nmol/L) compared with non-diabetic WT mice (5 nmol/L). No differences were observed between the vessels from non-diabetic Tg3 (7 nmol/L) and diabetic Tg3 (8 nmol/L) mice (Fig 1G).

Furthermore, PE produced a concentration dependent increase in contractile responses in aorta from all groups, which then reached a plateau level at 3 x 10^−6^ to 1 x 10^−5^ M. This general pattern was similar between the non-diabetic WT and Tg3 mice. Aortas from diabetic WT mice had higher contractile responses to PE (from 10^−7^ to 10^−5^ M), which were significantly above those of non-diabetic WT tissue. However, contractile responses in aortas of diabetic Tg3 mice were markedly lower than those of diabetic WT mice and were comparable to those in both non-diabetic control groups (Fig 1H). This data indicates that netrin-1 improves vascular endothelial function in diabetes.

#### 3.1.5 Netrin-1 stimulates production of nitric oxide (NO)

**Fig 2:**
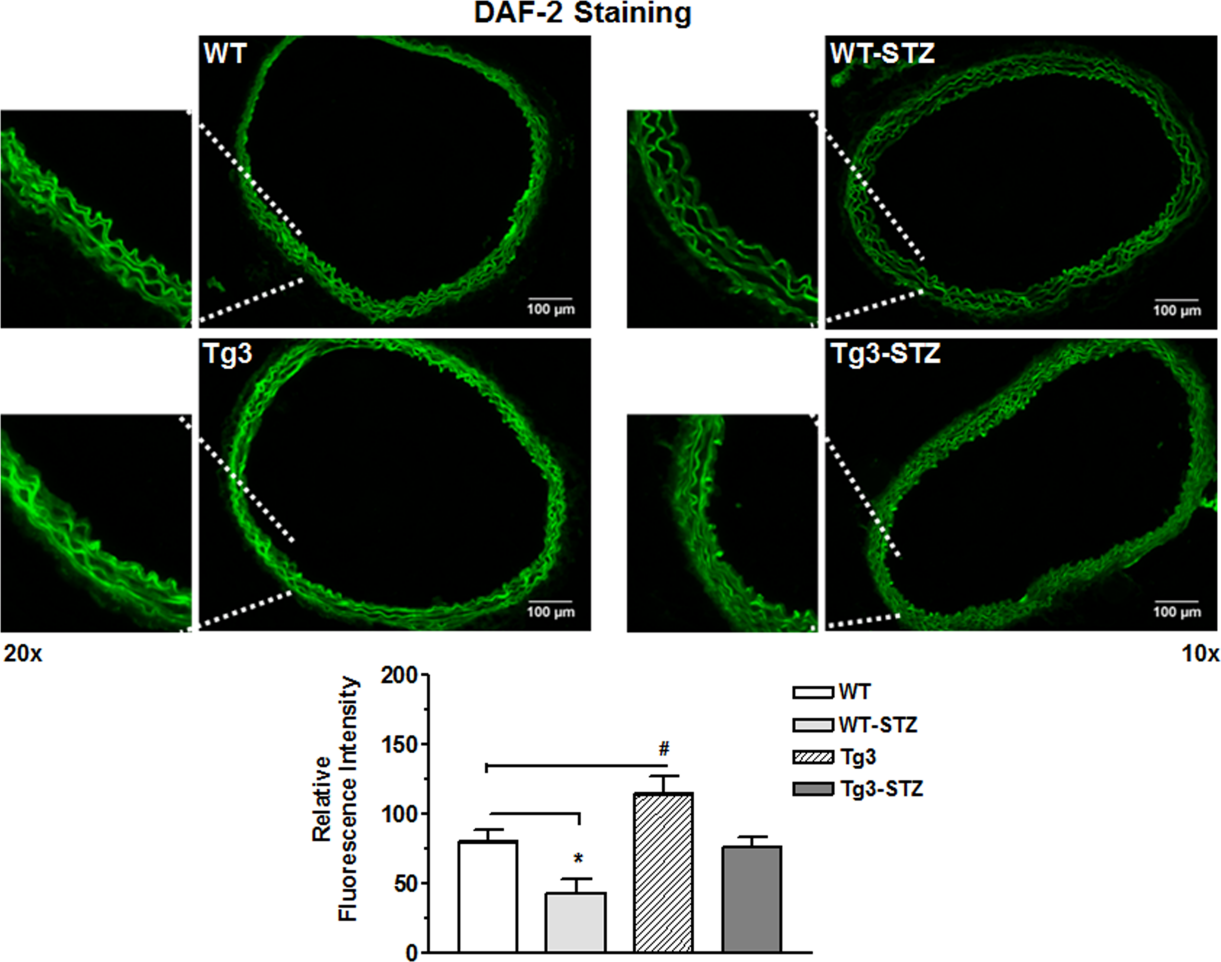
NO levels in aorta. Sections of aorta from non-diabetic and diabetic wild type (WT) and netrin-1 transgenic (Tg3) mice were reacted with 4, 5-diaminofluorescein (DAF-2). Representative DAF-2 fluorescence images were taken in aorta from all groups and analysis of fluorescence intensity normalized to non-diabetic WT group. Scale bar length is 100 μm. Data are mean ± S.E.M. of 4 experiments. *P < 0.05; ^#^*P* < 0.05, compared with non-diabetic WT group.

Diabetes is characterized by decreased NO bioavailability. We assessed whether Tg3 preserves NO levels in diabetes by using the fluorescent indicator 4,5-diaminofluorescein-2 diacetate (DAF-2; green color) Intensity of DAF-2 fluorescence was markedly reduced in vessels from the diabetic WT mice compared with those of the non-diabetic WT mice. Aortas from non-diabetic Tg3 mice exhibited much higher fluorescence intensity compared with non-diabetic WT mice. Diabetes-induced reduction in NO levels was less in Tg3 aortas than those of diabetic WT tissue (Fig 2). Additionally, NO levels in the diabetic Tg3 group were lower than in the non-diabetic Tg3 group. Pretreatment of aortic sections with NOS inhibitor L-NAME blocked DAF-2 fluorescence in control WT or Tg3 aortas (not shown). Our findings suggest that improved of endothelial function in diabetic Tg3 mice is associated with enhanced vascular NOS function and NO formation.

#### 3.1.6 Netrin-1 attenuates diabetes-induced oxidative stress

**Fig 3:**
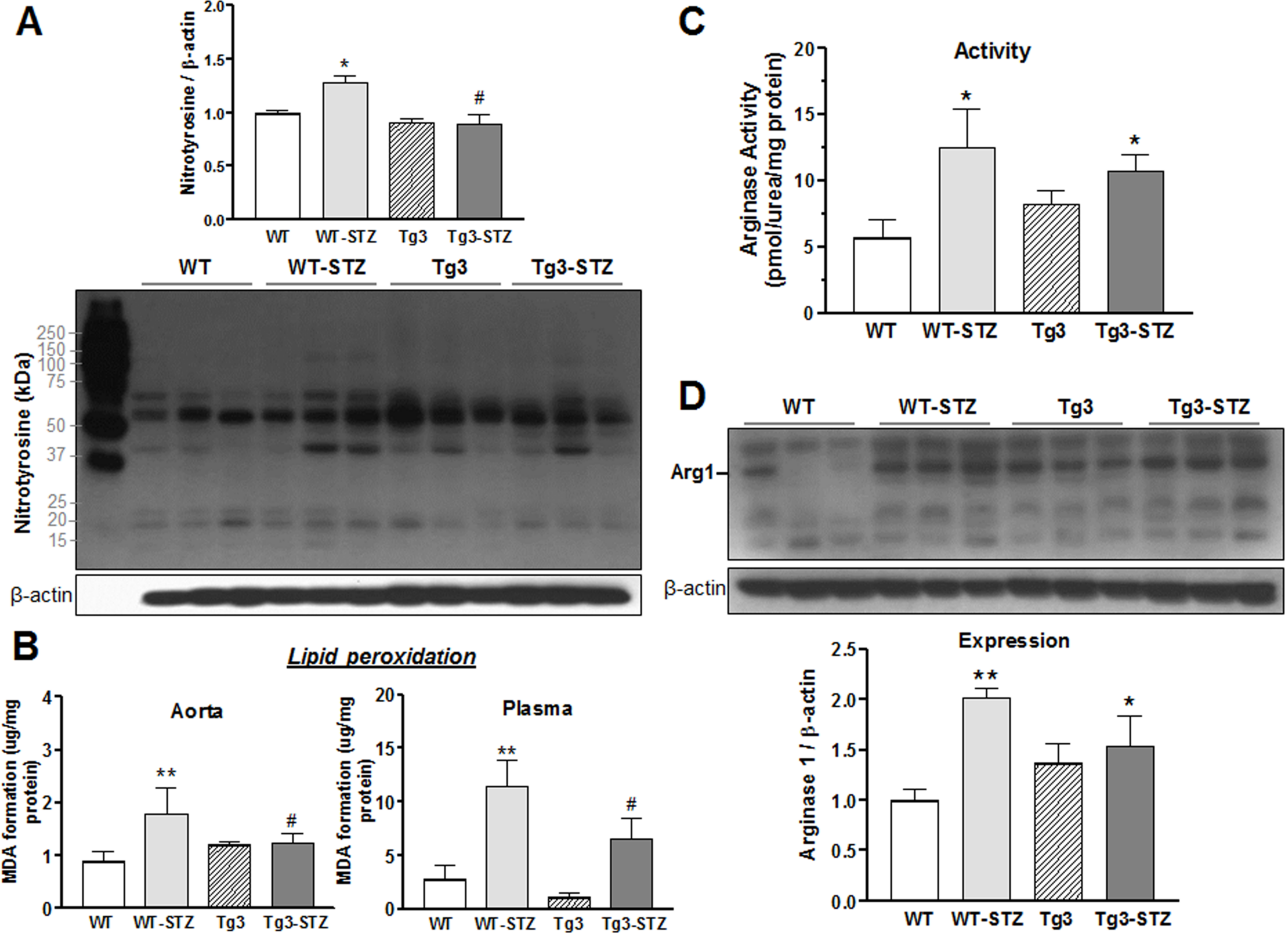
Nitrotyrosine content, lipid peroxidation and levels of arginase. (**A**) Formation of nitrotyrosine in aorta from non-diabetic and diabetic wild type (WT) and netrin-1 transgenic (Tg3) mice. (**B**) Malonaldehyde (MDA) formation was estimated by thiobarbituric acid (TBAR) assay in aorta and plasma from non-diabetic and diabetic WT and Tg3 mice. Vascular (**C**) arginase activity and (**D**) expression of arginase 1 (Arg1) were determined in aortas from non-diabetic and diabetic WT and Tg3 mice. Protein expression was normalized by β-actin levels and expressed as the fold change of non-diabetic WT group. Results are representative of 6 separate experiments. *P < 0.05; **P < 0.01; compared with non-diabetic WT group; ^#^*P* < 0.05, compared with diabetic WT group.

Increased oxidative stress as assessed by superoxide production, nitrotyrosine content, and indices of lipid peroxidation are characteristic of diabetes. To explore the role of oxidative stress in endothelium-protective action of netrin-1, we examined content of aortic nitrotyrosine and aortic and plasma malondialdehyde (MDA) in non-diabetic and diabetic WT and Tg3 mice. Superoxide anion (O_2_^∙−^) combines with nitric oxide (NO) rapidly to form a highly reactive species, peroxynitrite (ONOO^−^), which nitrates tyrosine residues in proteins. Thus nitrotyrosine is a biomarker for ONOO^−^. MDA is a stable metabolite of the ROS-mediated lipid peroxidation cascade and serves as an index of oxidative stress. Diabetic WT mice exhibited significant increases in nitrotyrosine and MDA content as compared with the non-diabetic WT group. Both indicators were lower in diabetic mice overexpressing netrin-1 as compared with diabetic WT mice (Fig 3A and 3B), suggesting that the endothelium-protective effect of netrin-1 involves inhibition of oxidative stress.

**Fig 4:**
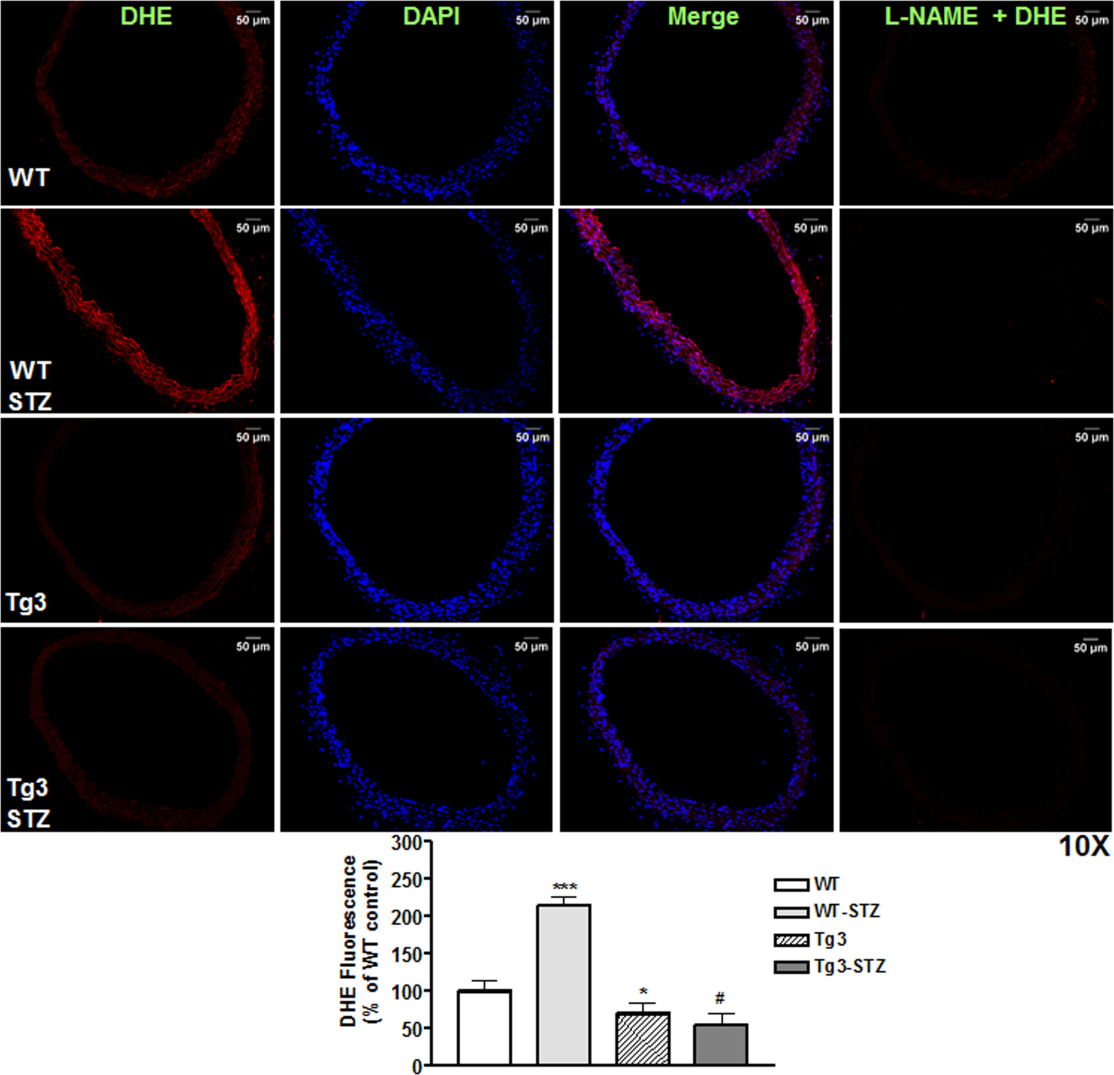
Reactive oxygen species formation in aorta. Sections of aorta from non-diabetic and diabetic wild type (WT) and netrin-1 transgenic (Tg3) mice were stained with DHE (marker of reactive oxygen species). DHE-stained signals are in red, DAPI-stained nuclei are in blue, and the merged signals are in purple. Mean DHE fluorescent intensity levels in aortas among groups. Scale bar length is 50 μm. Data are mean ± S.E.M. of 4–5 experiments. *P < 0.05; ***P < 0.001; compared with non-diabetic WT group; ^#^*P* < 0.001, compared with diabetic WT group.

Additionally, vascular superoxide production was determined using DHE imaging (red color) of mouse aortic sections. Fig 4 shows representative images of all groups. Aorta sections of diabetic WT mice had higher DHE signal intensity compared with the non-diabetic WT mice. DHE fluorescence was not different between aortas from non-diabetic WT and Tg3 mice. Importantly, aortic sections from diabetic mice overexpressing netrin-1 had markedly lower DHE signal intensity compared to non-diabetic WT aortas. Inhibition of the fluorescence by L-NAME (100 μmol/L) identified NOS as the main source of ROS in the aortas of diabetic WT mice.

#### 3.1.7 Netrin-1 does not prevent diabetes-induced increases in arginase activity and arginase 1 expression

Excessive activity of vascular arginase, an enzyme that competes with eNOS for their common substrate L-arginine, is observed in diabetes. We assessed whether the protective effect of netrin-1 in diabetes-induced VED involves down-regulation of expression and activity of arginase. We measured vascular arginase activity through urea production and its expression by western blot as reported previously [23]. We observed a 2.2-fold increase in arginase activity in diabetic WT aortas compared with non-diabetic WT tissue (Fig 3C). Arginase activity in non-diabetic aortas of Tg3 and WT mice was not different. Aortas from diabetic Tg3 mice exhibited increases in arginase activity levels similar to those in diabetic WT aorta (Fig 3C). Additionally, expression of arginase 1 (Arg1) was increased by 2-fold in aorta from diabetic WT vs non-diabetic WT tissue, while arginase 2 (Arg2) was not significantly altered as compared with non-diabetic WT mice (data not shown). Arg1 levels in non-diabetic WT and Tg3 mice were not significantly different. However, elevated Arg1 protein levels were observed in diabetic Tg3 aortas compared to non-diabetic WT tissues (Fig 3D). These data indicate that overexpression of netrin-1 does not prevent diabetes-induced elevation of vascular arginase expression/activity.

#### 3.1.8 Netrin-1 suppresses diabetes-induced inflammatory and apoptotic markers

**Fig 5:**
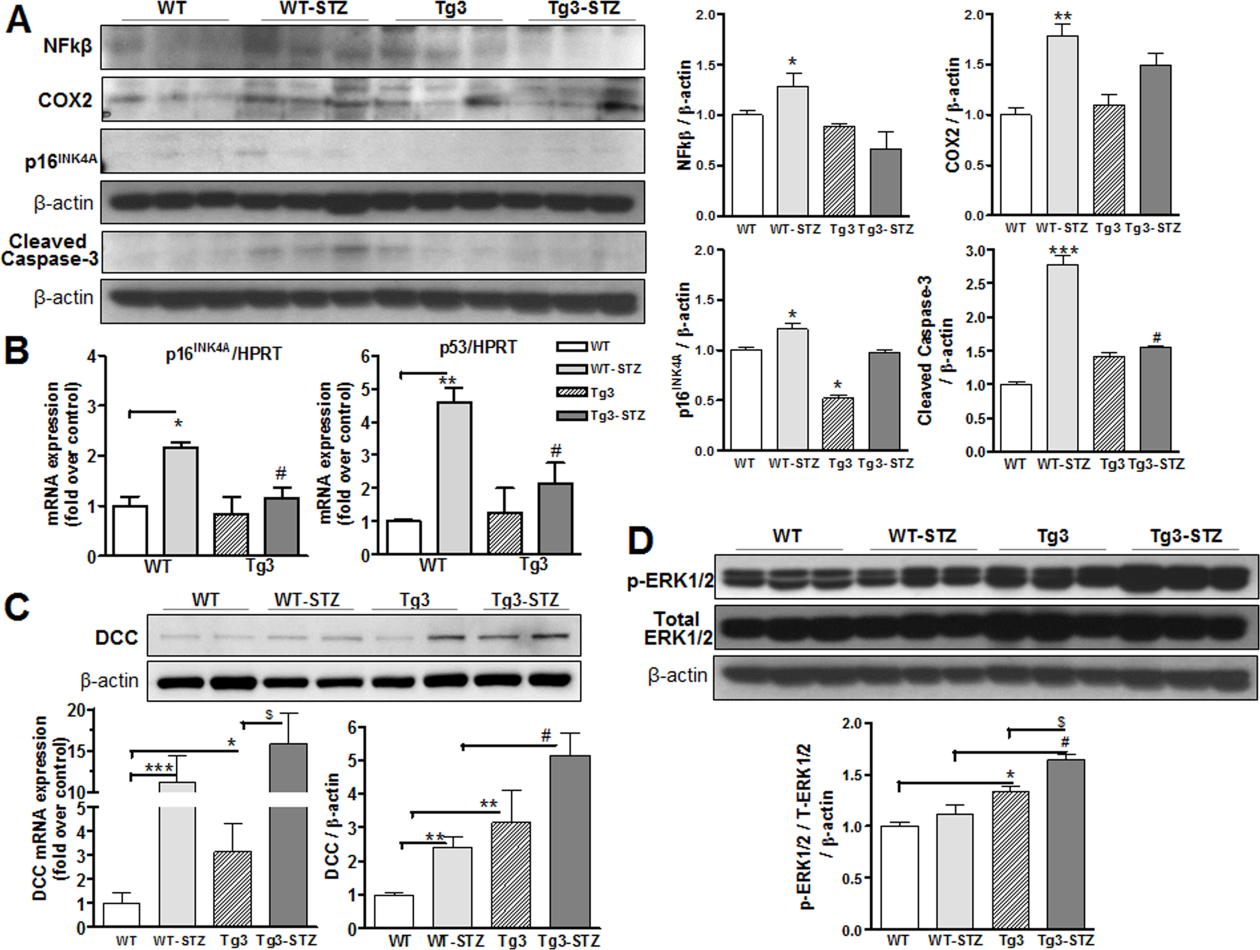
Molecular signaling in response to diabetes. **(A)** Protein expression of NFκB, COX2, p16^INK4A^ and cleaved caspase-3 were measured in aortas from non-diabetic and diabetic wild type (WT) and netrin-1 transgenic (Tg3) mice. Protein expression was normalized by β-actin levels and expressed as the fold change of the non-diabetic WT group. (**B**) Real-time RT-PCR analysis of p16 and p53 in aortas from non-diabetic and diabetic WT and Tg3 mice. **(C)** RT-PCR analysis and protein levels of DCC in aortas from non-diabetic and diabetic WT and Tg3 mice. **(D)** Phosphorylated-and total ERK1/2 levels were measured in aortas among groups, and then normalized by β-actin and expressed as the fold change of the non-diabetic. Results are representative of 4–5 separate experiments. *P < 0.05; **P < 0.01; ***P < 0.001, compared with non-diabetic WT group; ^#^*P* < 0.05, compared with diabetic WT group; ^$^*P* < 0.05, compared with non-diabetic Tg3 group.

Increased levels of systemic markers of inflammation and apoptosis are seen in diabetes [24]. We assessed whether the protective effect of Tg3 mice in diabetes-induced VED is mediated by reducing expression of NFκB and COX2 (inflammatory markers) and levels of p16^INK4A^ and cleaved caspase-3 (apoptotic markers). We observed that diabetes markedly increased NFκB and COX-2 expression in WT mice, but expression levels were significantly lower in mice overexpressing netrin-1. Further, expression levels of p16^INK4A^ and cleaved caspase-3 were markedly increased in diabetic WT mice, but expression of these factors were less in Tg3 mice (Fig 5A). Additionally, mRNA levels of p16 and p53 were markedly increased in aortas from diabetic mice compared with those of non-diabetic tissues, whereas aortas from diabetic Tg3 mice did not show significant changes compared with non-diabetic Tg3 samples (Fig 5B). These findings suggest that netrin-1 may improve vascular function in diabetes by reducing levels of inflammation and apoptotic markers.

#### 3.1.9 Netrin-1 and its effect on DCC/ERK1/2 pathway

To characterize the signaling mechanism underlying netrin-1’s effects on vascular function, we assessed levels of its receptor ‘deleted in colorectal cancer’ (DCC) and components of its downstream pathway in aortic tissues from non-diabetic and diabetic WT and Tg3 mice. Expression levels of DCC in aortic samples were determined by mRNA and western blot. Increased levels of DCC mRNA and protein were observed in aortas of non-diabetic Tg3 mice compared with the non-diabetic WT mice. Surprisingly, diabetes also markedly increased DCC mRNA and protein expression in aortas of WT mice compared those of non-diabetic WT mice. Further, diabetic Tg3 aortas had higher levels of DCC mRNA and protein expression than non-diabetic Tg3 or diabetic WT tissue (Fig 5C).

Since our data show increased vascular NO levels in mice overexpressing netrin-1, we also determined whether netrin-1 effects are associated with mitogen-activated protein kinase ERK1/2 activation. Earlier studies have shown that activated ERK1/2 is involved with netrin-1-induced stimulation of NO production of endothelial cells [10]. We found that aortas of mice overexpressing netrin-1 had significantly higher levels of phosphorylated ERK1/2 to total ERK1/2 content compared with those of non-diabetic WT mice (Fig 5D). Protein levels of phospho-ERK1/2 were not different between aortas from diabetic and non-diabetic WT mice. However, aortic levels of phosphorylated ERK1/2 were higher in diabetic Tg3 compared with non-diabetic Tg3 mice. Our findings suggest that the vaso-protective effects of netrin-1 overexpression occur through the DCC/ERK1/2 pathway.

### 3.2 Cellular studies

#### 3.2.1 Netrin-1 and DCC expression in endothelial cells

**Fig 6:**
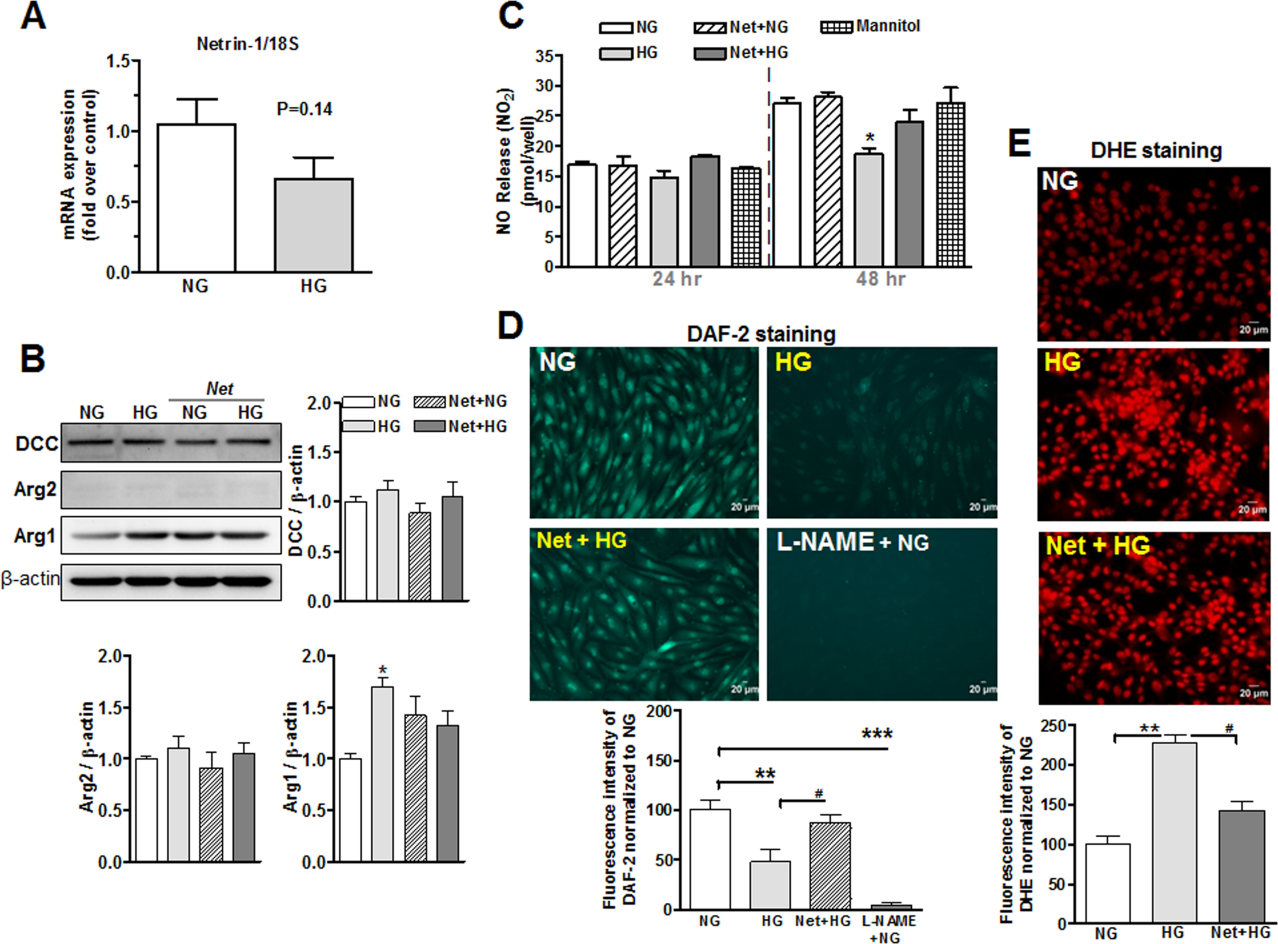
Levels of netrin-1, DCC, arginase, NO and ROS in endothelial cells. (**A**) Real-time RT-PCR analysis of netrin-1 in bovine aortic endothelial cells (BAEC) after exposure to normal glucose (NG, 5.5 mmol/L) or high glucose (HG, 25 mmol/L) for 48 hrs. **(B)** Protein levels of DCC, arginase 1 (Arg-1) and arginase 2 (Arg-2) in BAECs exposed to NG or HG media in the absence or presence of netrin-1 (100 ng/ml) for 48 hr. (**C**) Cumulative NO production measured by chemiluminescence after exposure to NG, HG or mannitol media in BAEC pre-treated with netrin-1 (100 ng/ml) for 24 and 48 hrs. (**D**) NO-specific fluorescence of DAF-2 and (**E**) oxidative stress-specific fluorescence of DHE in BAEC exposed to NG, HG and HG + netrin-1 for 48 hr. Scale bar length is 20 μm. Values were normalized to NG group. Data are the mean ± S.E.M. of 4–6 experiments. *P < 0.05; **P < 0.001; ***P < 0.001, compared with NG group; ^#^*P* < 0.05, compared with HG group.

Since hyperglycemia is considered a primary cause of diabetic VED, we examined levels of netrin-1 and its downstream pathway in BAECs exposed to normal glucose (NG, 5.5 mmol/L), high glucose (HG, 25 mmol/L) or mannitol (5.5 mmol/L D-Glucose + 19.5 mmol/L mannitol) media for 48 hrs. We observed that netrin-1 mRNA levels in BAECs treated with either NG or HG media were not significantly different (P = 0.14; n = 6, per group) (Fig 6A). Cells treated with mannitol exhibited mRNA netrin-1 levels that were similar to those of NG-treated cells (not shown).

To determine levels of the receptor for netrin-1, we measured protein expression of DCC in endothelial cells. DCC protein expression levels were not different between the NG and HG groups. Pretreatment of cells exposed to NG or HG with netrin-1 did not alter DCC levels (Fig 6B).

#### 3.2.2 Netrin-1 does not limit elevation of arginase activity/expression in BAEC, but prevents high glucose (HG)-induced reduction of NO levels and elevation of ROS formation

Since increased arginase activity is associated with NOS uncoupling [6], we assessed levels of arginase activity/expression in BAEC pre-treated with netrin-1 under HG or NG media. Exposure of BAECs to HG (48 hr) increased Arg1 expression by 70% compared with those of NG group. It is important to note that, this elevation was not significantly altered by pretreatment with netrin-1 in HG-treated cells (Fig 6B) even though values tended to be lower. Further, Arg2 levels were not altered in NG and HG treated cells with or without pretreatment with netrin-1.

We noted that cells exposed to HG reduced the accumulation of NO in culture medium by 32% at 48 hr *vs* NG (Fig 6C). No differences in NO levels were observed at 24 hr among the groups. Pretreatment with netrin-1 (100 ng/ml) partially prevented HG-induced reduction of NO levels to only 11% at 48 hr. Further, exposure of cells to mannitol did not alter NO levels at either 24 or 48 hr compared with NG-treated cells.

Measurement of DAF-2 fluorescence showed that HG (48 hr) markedly decreased intracellular NO levels by ~52% compared with NG group. However, pretreatment of BAECs with netrin-1 blunted this HG-induced reduction of NO levels to only 14%. Treatment of cells with L-NAME (100 μmol/L, 30 min) prevented DAF-2 fluorescence, indicating that eNOS is the source for NO production (Fig 6D). Also, BAEC exposed to HG (48 hr) exhibited markedly increased superoxide generation (DHE fluorescence) compared with cells exposed to NG medium. However, pretreatment of BAECs with netrin-1 blocked the HG-induced elevation of ROS (Fig 6E).

#### 3.2.3 Netrin-1 and its effect on apoptosis in BAECs

**Fig 7:**
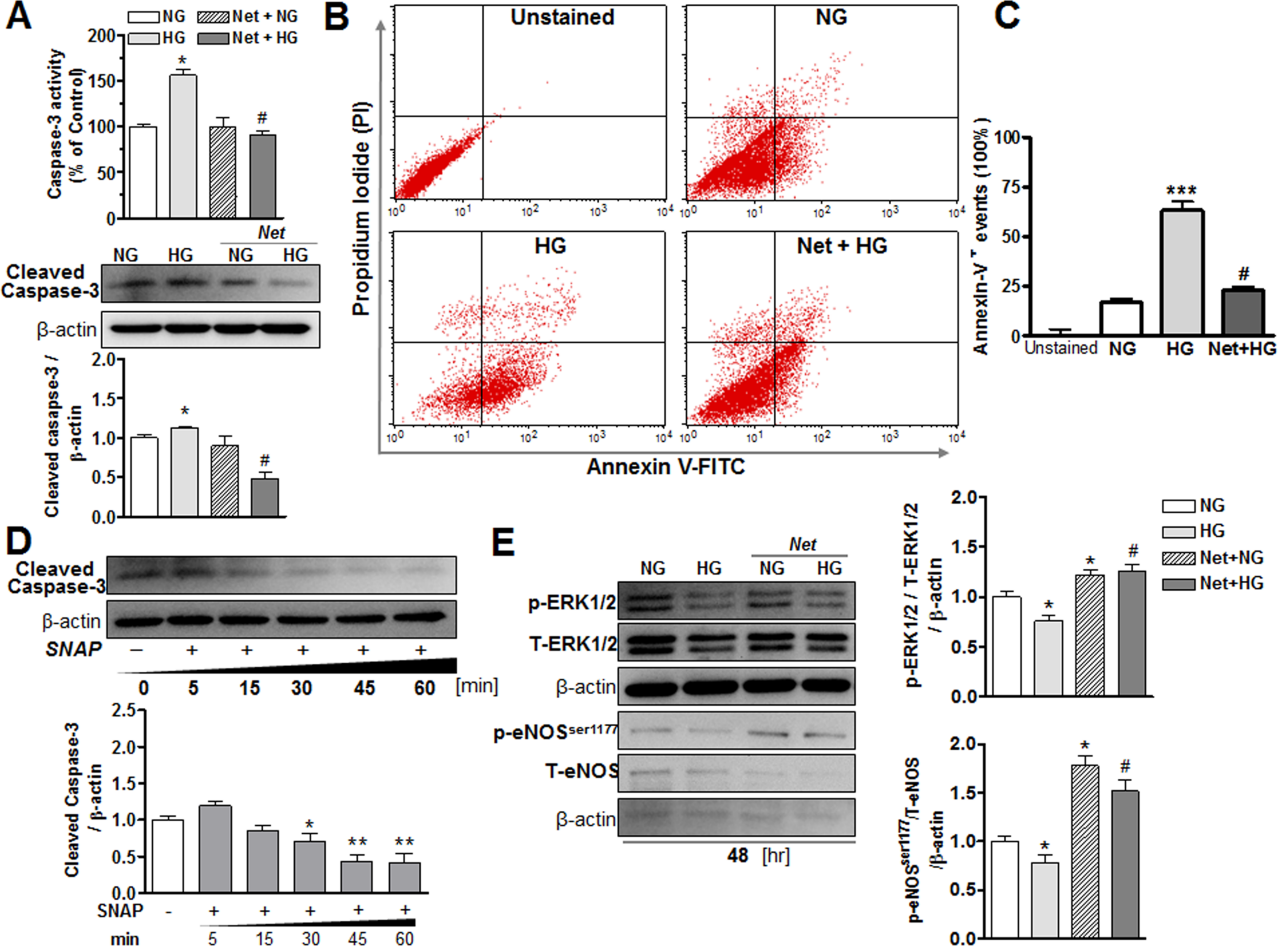
Molecular signaling in response to high glucose in endothelial cells. (**A**) Activity and expression of caspase-3 in bovine aortic endothelial cells (BAECs) exposed to normal glucose (NG, 5.5 mmol/L) or high glucose (HG, 25 mmol/L) media in the presence or absence of netrin-1 (100 ng/ml) for 48 hr. **(B)** Flow cytometric analysis of apoptosis in BAECs exposed to NG and HG media for 48 hrs. Cells were exposed to netrin-1 (100 ng/ml) before exposure with HG media and **(C)** quantification of the annexin V^+^ cells ratio was performed in each group and expressed as percentage of NG group. **(D)** Protein levels of cleaved caspase-3 in NG-treated cells after exposure to a NO donor, SNAP (10 μmol/L), at different time points (5, 15, 30, 45, or 60 min). **(E)** Expression of p-ERK1/2, total ERK1/2, p-eNOS^ser1177^ and total eNOS in BAEC treated with NG or HG with or without netrin-1. p-ERK1/2 or p-eNOS^Ser1177^ levels were normalized to total ERK1/2 or total eNOS expression, and then normalized by β-actin levels and expressed as the fold change of NG group. Data are mean ± S.E.M. of 4–6 experiments. *P < 0.05; **P < 0.01; ***P < 0.001, compared with NG group; ^#^*P* < 0.05, compared with HG group.

To determine whether netrin-1 affects hyperglycemia-induced apoptosis in endothelial cells, measurements of activity and expression of caspase-3 and flow cytometric analysis of annexin-V and propidium iodide (PI) were made. BAECs exposed to HG (48 hr) exhibited markedly increased caspase-3 activity by ~60% *versus* the NG group at 48 hr. Pretreatment with netrin-1 did not alter either activity or protein levels of caspase-3 in the NG group, but markedly reduced activity and expression of caspase-3 in HG-treated cells (Fig 7A). Further, HG-exposed cells displayed a significant increase in apoptotic (annexin-V positive) cells compared with NG cells, and netrin-1 treatment of HG cells reduced this effect (Fig 7B). Quantification of the annexin-V^+^ cells (expressed as percentage) for each group is represented in Fig 7C.

#### 3.2.4 NO donor and its effects in cleaved caspase-3 protein levels in BAECs

To examine the effects of NO on cleaved caspase-3 expression, we exposed NG-treated BAECs to the NO donor S-nitroso-N-acetylpenicillamine (SNAP, 10 μmol/L) over 60 min. We observed that SNAP markedly blunted levels of cleaved caspase-3 in a time dependent manner beginning at 30 (by -29%), 45 (by -56%) and 60 (by -58%) min of treatment (Fig 7D). These data suggest that NO may facilitate and preserve endothelial function by decreasing cell apoptosis.

#### 3.2.5 Netrin-1 and its effect in ERK1/2 and eNOS expression in BAECs

To determine if the downstream signaling of netrin-1 involves ERK1/2 and activation of eNOS, we assessed levels of phosphorylated and total ERK1/2 and eNOS in BAECS exposed to NG or HG media. BAECs exposed to HG for 48 hr exhibited significantly reduced levels of phosphorylated ERK1/2 by 25% compared with those of exposed with NG. Pretreatment of NG cells with netrin-1 increased their levels of phospho-ERK1/2. Netrin-1 also prevented the reduction caused by HG (Fig 7E). Additionally, HG decreased levels of p-eNOS^ser1177^ by 30% compared to NG group. However, netrin-1 pretreatment markedly increased p-eNOS^ser1177^ in NG-treated cells and prevented HG-induced reduction of p-eNOS^Ser1177^ levels (Fig 7E). Our findings suggest that in hyperglycemia, netrin-1 maintains levels of p-eNOS^ser1177^ through activation of p-ERK1/2.

## Discussion

This study provides strong evidence of a vaso-protective action of netrin-1 in diabetes. We projected that overexpression of netrin-1 in mice may increase NO production through DCC/ERK1/2 pathway, and via this action could serve to enhance endothelial cell function. We observed that in the diabetic vasculature, overexpression of netrin-1: 1) prevents impairment of endothelial function, 2) reduces vascular and systemic oxidant generation, 3) elevates NO levels through a DCC—ERK1/2 pathway, and 4) suppresses inflammatory and apoptotic processes. Despite these beneficial effects, overexpression netrin-1 had no impact on weight gain, glucose metabolism or insulin levels. Further, studies with BAECs exposed to high glucose (HG) showed reduced levels of netrin-1. These effects were associated with decreased NO production, increased reactive oxygen species (ROS), enhanced caspase-3 activity and expression, and decreased levels of phosphorylated ERK1/2 and eNOS^ser1177^. Treatment of BAECs with netrin-1 prevented HG-induced elevation of oxidative stress and apoptosis (measured by caspase-3 activity/expression and annexin-V^+^ cells), maintained NO levels, restored ERK1/2 activity and increased p-eNOS^ser1177^ levels. Thus, our results indicate that enhancement of netrin-1 effects in hyperglycemia/diabetes may represent a therapeutic means for amelioration of endothelial cell dysfunction.

In addition to its importance in neuronal development, netrin-1 has broad relevance to many other biological processes, including cell migration, proliferation and the determination of cell fate [25–27]. Evidence indicates that netrin-1 is expressed in the vascular endothelial cells, and that its expression is largely reduced during acute inflammation [28]. Netrin-1 also strongly reduces leukocyte recruitment into the vascular wall in atherosclerosis and lack or inhibition of netrin-1 by proatherogenic factors have shown to increase leukocyte adhesion to the endothelium [29]. We observed that netrin-1 is expressed in the vascular aorta and BAECs, and that its expression is markedly decreased by diabetes/hyperglycemia. Reduction of vascular netrin-1 levels in diabetic WT mice appears to be involved in impaired endothelial function because overexpression of netrin-1 in mice largely prevents diabetes-induced endothelial impairment. Although the signaling mechanism of netrin-1 in the vascular endothelium is not well known, our data show that activation of DCC/ERK1/2 pathway by netrin-1 leads to elevation of NO production in the endothelial cells.

The effects of netrin-1 depend on specific interactions with its receptors such as DCC and UNC5 family members, which are differentially expressed in various tissues [12,30–32]. Netrin-1 receptors have been found in stem, epithelial and endothelial cells [33,34], suggesting influence of netrin-1 on morphogenesis [35], control of apoptosis [36] and angiogenesis [17]. Interaction of netrin-1 with the DCC receptor is critical in several cellular processes including cell survival, proliferation and migration of human cerebral endothelial cells, tissue organization, cardiac protection, angiogenesis and cancer [11,18,34,37–39]. However, in the absence of netrin-1, DCC can inhibit cellular survival and elicit an apoptotic signal through activation of caspase-3 [40]. Thus, cells expressing DCC receptor are dependent on netrin-1 in the extracellular environment to survive. Our results show that DCC mRNA and protein are expressed in mouse aorta, and increased in aortas of Tg3 non-diabetic or diabetic mice compared with those of WT mice. Interestingly, enhanced DCC levels (mRNA and protein) were observed in aortas of diabetic compared to those of non-diabetic WT mice. We speculate that the enhanced levels of DCC and netrin-1 in Tg3 mice activate ERK1/2 and increase eNOS activation. Suppression of netrin-1 and elevation of DCC levels during diabetes allows DCC to promote apoptosis. We observed that exposure of cells to HG did not alter levels of DCC compared with those of NG group, but that treatment of HG-exposed cells with netrin-1 increased ERK1/2 activation.

Endothelial dysfunction is a critical feature of diabetes, and includes impaired vasorelaxation, NOS uncoupling with elevation of oxidative stress, reduced vascular NO production, vascular thickening, and endothelial apoptosis. eNOS activity and NO bioavailability are regulated by several mechanisms including transcription and post-transcription and the reduction of NO by ROS-mediated quenching [41]. We observed increased NO levels in aortas from diabetic Tg3 *vs* WT mice. The reduction in NO levels in HG-treated BAEC was prevented by treatment with netrin-1. Also, the increased levels of HG-induced superoxide in endothelial cells and of superoxide, nitrotyrosine, and lipid peroxides in aorta of diabetic WT mice were largely attenuated by treatment with netrin-1 or in Tg3 mice. Previous studies have observed that binding of netrin-1 to DCC in endothelial cells leads to activation of ERK1/2, with subsequent phosphorylation and activation of eNOS [10,11]. Data from our endothelial cell studies support our *in vivo* observations in which treatment of HG-exposed BAECs with netrin-1 increased activation of ERK1/2 and phospho-eNOS^ser1177^ followed with enhanced NO production. Our findings clearly indicate that netrin-1 enhances eNOS activity by a posttranslational mechanism and reduces vascular ROS in diabetes/hyperglycemia.

Many studies have reported that a cellular deficiency of NOS substrate L-arginine can cause VED by uncoupling eNOS. Enhanced arginase activity has been strongly implicated in the depletion of L-arginine, leading to NOS dysfunction in diabetes [42]. As expected, enhanced arginase activity and Arg1 levels were observed in diabetic WT mice and HG-treated BAECs compared with their respective controls. These elevations of arginase activity/expression were not different from those observed in diabetic Tg3 mice or in BAEC pretreated with netrin-1 before HG exposure. Further, levels of arginase activity in aortas of non-diabetic WT mice were similar to those of Tg3 mice. An important question arising from our findings is why an increase in netrin-1: 1) reduces ROS formation; 2) increases NO levels, and 3) prevents vascular dysfunction in diabetic mice even though vascular levels of arginase are elevated, which is expected to do the opposite. A possible explanation is that netrin-1-induced enhancement of NOS activity could limit arginase by providing high intracellular levels of hydroxyl-L-arginine (NOHA) and citrulline, both of which inhibit arginase [43,44].

In addition to netrin-1 causing activation of eNOS, prior studies have shown that it is a key regulator of inflammation and apoptosis in ischemic stroke, hypoxic mesenchymal stem cells and diabetic nephropathy [14,15,20]. It is important to note that plasma netrin-1 levels has been shown to be markedly decreased in patients with diabetes, and this effect was negatively associated with insulin resistance and glucose homeostasis [45]. In contrast, long term netrin-1 treatment in mice showed a preventive role against high-fat diet/STZ-induced diabetes through maintained islet insulin secretion and reduction of vascular inflammation [46]. Overexpression of netrin-1 or administration of recombinant netrin-1 reduced cell injury and restored impaired-angiogenesis in vascular endothelial cells [47].

Hyperglycemia is known to activate NFκB, a transcription factor involved in the activation of a wide variety of genes including cytokines, chemokines, iNOS and COX-2 [48–50]. COX-2 is an inducible isoform that participates in pro-inflammatory responses and its enhancement has tissue specific consequences, which lead to structural and functional changes in diabetes [51]. Down-regulation of netrin-1 during organ injury may exacerbate inflammation [52,53]. Overexpression of netrin-1 can suppress kidney inflammation and apoptosis *in vivo* [28,52,54]. Our data suggest that netrin-1 decreases inflammation through inhibition of NFκB activation, attenuates diabetes-induced COX-2 expression and suppresses apoptotic processes by reducing p16^INK4A^ and caspase-3 activity and expression. Additionally, exposure of BAECs to a NO donor markedly reduced levels of cleaved-caspase-3, suggesting that NO preserves endothelial function and decreases apoptosis. A recent study showed that glucose induced degradation of IkBα and enhanced translocation of p65-NFκB from the cytoplasm to the nucleus in TKPTS cells, and that netrin-1 suppressed these changes [20]. We speculate that suppression of COX-2 in Tg3 diabetic tissue may occur through diminished translocation of p65- NFκB protein to the nucleus. Although we observed that transgenic overexpression of netrin-1 in mice promotes healthy endothelial cell function and suppress inflammatory and apoptotic processes in diabetes, further studies are needed to determine whether suppression of inflammation by netrin-1 occurs through IkBα-mediated inhibition of NFκB in diabetes.

Diabetes has been implicated in the structural and functional alteration of several organ systems, including the central nervous system (CNS) and blood brain barrier (BBB) [55,56]. Integrity of BBB function depends on tight junction-associated proteins in the vascular endothelium of the brain. Recent reports have indicated that netrin-1 treatment promotes the expression of tight junction proteins and enhanced BBB function leading to reduced brain lesions, decreased levels of inflammatory markers and delayed onset in multiple sclerosis disease [57,58]. However, since the role of netrin-1 in diabetes-induced BBB dysfunction has not been well documented, future studies are necessary to determine if increased netrin-1 levels represent a therapeutic strategy to prevent BBB disruption and CNS inflammation.

In summary, our study clearly shows that diabetes decreases vascular netrin-1 levels and that overexpression of netrin-1 attenuates diabetes-induced VED. These effects appear to involve elevation of NO levels through DCC/ERK1/2 pathway, reduction of vascular ROS production and nitro-oxidative stress, and suppression of inflammatory and apoptotic processes. We conclude that enhancement of netrin-1 may represent a useful therapeutic means for restoring vascular function in diabetes and potentially other diabetic complications.
